# Acetaldehyde and methylglyoxal: comparative analysis of toxic electronic cigarette degradation products in 3D and 2D exposure systems using human bronchial epithelial models

**DOI:** 10.3389/ftox.2025.1624794

**Published:** 2025-09-30

**Authors:** Man Wong, Teresa Martinez, Nathan Hendricks, Prue Talbot

**Affiliations:** ^1^ Department of Molecular, Cell and Systems Biology, University of California, Riverside, Riverside, CA, United States; ^2^ UCR Proteomics Core, University of California, Riverside, Riverside, CA, United States

**Keywords:** proteomics, aldehydes, acetaldehyde, methylglyoxal, bronchial epithelium, electronic cigarettes

## Abstract

**Background:**

Acetaldehyde and methylglyoxal are structurally related aldehydes produced by thermal degradation of the electronic cigarette (EC) solvents, propylene glycol and glycerin. Despite their presence in EC aerosols, the biological effects of these aldehydes when inhaled during vaping are largely unknown.

**Methods:**

Three-dimensional (3D) human bronchial epithelial tissues (EpiAirway™) were exposed at the air liquid interface (ALI) to aerosols containing acetaldehyde or methylglyoxal at concentrations relevant to human vaping. PBS-exposed tissues served as controls. Comparative proteomic analyses were performed to assess global alterations in protein expression. Based on proteomics data, concentration-response experiments were conducted using BEAS-2B bronchial epithelial cells to evaluate reactive oxygen species, mitochondrial function, and cytoskeletal integrity.

**Results:**

ALI exposure to acetaldehyde or methylglyoxal resulted in 79 and 76 differentially expressed proteins (DEPs), respectively, with 51 overlapping proteins exhibiting similar fold change directionality. Ingenuity Pathway Analysis (IPA) Toxicity Lists identified key affected pathways, including mitochondrial dysfunction, fatty acid metabolism, G2/M DNA damage checkpoint regulation, and mitochondrial biogenesis. Gene Ontology (GO) ontology analysis revealed substantial overlap in affected biological processes and cellular components. Findings were further supported and expanded in BEAS-2B cell concentration-response assays, which confirmed mitochondrial impairment, elevated ROS levels, and disrupted cytoskeletal organization. Notably, TRPM8 inhibition attenuated methylglyoxal-induced mitochondrial dysfunction (MTT assay), while both TRPM8 and TRPA1 inhibition partially rescued actin depolymerization.

**Conclusion:**

Brief ALI exposure of EpiAirway™ tissues to vaping-relevant concentrations of acetaldehyde or methylglyoxal altered the bronchial epithelial proteome. Complementary concentration-response experiments with submerged BEAS-2B cells confirmed and extended the proteomics data. While both aldehydes exhibited similar proteomic and functional impacts, methylglyoxal was effective at substantially lower concentrations in all assays with some effects partially mediated via TRPA1 and TRPM8 channels.

## 1 Introduction

Potentially harmful aldehydes are present in EC aerosols, but little is known about their relative toxicities in persons who vape. This study focuses on acetaldehyde and methylglyoxal, two aldehydes commonly detected in both EC fluids and aerosols (Jensen et al., 2017; Ogunwale et al., 2017; Talih et al., 2017; Uchiyama et al., 2020; Azimi et al., 2021; Omaiye et al., 2024). Acetaldehyde is classified by the U.S. Environmental Protection Agency as a probable human carcinogen (Group 2B) (U.S. Environmental Protection Agency, 1988) and has been studied extensively in respiratory pathology due to its high concentrations in cigarette smoke and in its role in ethanol metabolism in the liver (Barry, 1988; Appelman et al., 1982; Kawano et al., 2004; Setshedi et al., 2010; Wyatt et al., 2012; Sapkota and Wyatt, 2015; Li et al., 2019). In addition to its carcinogenic potential, acetaldehyde contributes to the development of cardiovascular disease, non-cancerous lung disease (Sapkota and Wyatt, 2015; Chen et al., 2019), and reinforcement of nicotine addiction (Talhout et al., 2007; Cao et al., 2007).

Methylglyoxal, although less extensively studied in the context of respiratory toxicity, has been implicated in the pathogenesis of diabetes-related complications and various cancers (Matafome et al., 2013; Bellahcène et al., 2018; Bellier et al., 2019; Schalkwijk and Stehouwer, 2020). It is more chemically reactive than acetaldehyde and has the capacity to form advanced glycation end-products (AGEs) and adducts with DNA, proteins, and lipids, which contributes to its genotoxic and cytotoxic potential (Setshedi et al., 2010; Sapkota and Wyatt, 2015; Schalkwijk and Stehouwer, 2020; Oliveira et al., 2020; Chen et al., 2007; Nissen et al., 2022; Trellu et al., 2019). While both chemicals have been detected in EC aerosols (Jensen et al., 2017; Ogunwale et al., 2017; Talih et al., 2017; Uchiyama et al., 2020; Azimi et al., 2021; Omaiye et al., 2024), the biological effects of methylglyoxal on the respiratory epithelium of EC users have received limited attention, possibly due to its relatively low reported concentrations in cigarette smoke and EC aerosols to other aldehydes (Moree-Testa and Saint-Jalm, 1981; Uchiyama et al., 2013; Bekki et al., 2014; Uchiyama et al., 2016).

Most work on acetaldehyde and methylglyoxal in cigarette smoke and EC aerosols has focused on their identification and quantification, but not a side-by-side comparison of their toxicities. To address this gap, we tested the hypothesis that both aldehydes would elicit overlapping biological responses due to their similar structures, but methylglyoxal would be more potent due to its higher chemical reactivity. To evaluate this, EpiAirway™ tissues (MatTek), which model the structural and functional features of the human tracheobronchial epithelium, were exposed to acetaldehyde or methylglyoxal at the ALI using a VitroCell™ cloud chamber. This exposure system employs vibrating mesh technology that produces aerosols with minimal heat generation, thereby avoiding thermal decomposition artifacts typical of heated coil systems (Nair et al., 2020). Tissues were exposed to aerosols containing acetaldehyde, methylglyoxal, or PBS (control) at concentrations within the estimated exposure range for EC users. Methylglyoxal was delivered at a lower concentration, consistent with its typically lower reported levels in EC aerosols as shown in Supplementary Tables S1, S2.

Following exposure, proteomic profiling of tissue lysates was conducted to obtain a global overview of differentially expressed proteins. Enrichment analysis of the proteomics data using Gene Ontology (GO) and Ingenuity Pathway Analysis (IPA) software identified several dysregulated pathways in response to both acetaldehyde and methylglyoxal. Mitochondria, oxidative stress, and the cytoskeleton were identified in the enrichment analysis as major targets of the aldehydes. These findings were further supported using concentration-response experiments with BEAS-2B cells in submerged monolayer cultures. Since prior studies demonstrated that environmental chemicals, including acetaldehyde and methylglyoxal, can activate members of the transient receptor potential (TRP) channel family (Zhang M. et al., 2023; Steib et al., 2025; Chung et al., 2019; Johnson et al., 2022; Effah et al., 2022; Bang et al., 2007; Andersson et al., 2013; Ciobanu et al., 2016), we also determined if the observed effects on mitochondria and the cytoskeleton were mediated by TRPA1 and TRPM8 channels.

## 2 Methods

### 2.1 EpiAirway™ tissue culture

EpiAirway™ (AIR-100-PE12), a ready-to-use 3-D mucociliary tissue model of normal human respiratory epithelium, was prepared and used for exposures, as described previously (Nair et al., 2020; Phandthong et al., 2024). EpiAirway™ tissue, which is derived from tracheal/bronchial cells and cultured at the ALI, recapitulates the *in vivo* phenotype, and is cultured at the ALI. EpiAirway™ tissues were shipped on cell culture inserts by MatTek Corp (Ashland, MA) in agarose shipping medium. Upon receipt, tissues were activated by replacing shipping medium with 750 uL of EpiAirway™ Maintenance Medium (AIR-100-MM), then incubated overnight at 37 °C and 5% CO_2_/95% relative humidity, according to the MatTek protocol.

### 2.2 VITROCELL™ cloud chamber exposures of EpiAirway™ tissues

Exposure of EpiAirway™ to acetaldehyde or methylglyoxal aerosols was done in a VitroCell cloud chamber (VITROCELL^®^ 12/12 base module, Walkirch, Germany) (Nair et al., 2020; Phandthong et al., 2024). EpiAirway™ maintenance medium was equilibrated to 37 °C in each well of the cloud chamber for 15 min before exposure. Aerosols were generated using an Aerogen vibrating mesh nebulizer (AG-AL1100, San Mateo, California). 200 μL of either PBS- (phosphate buffered saline minus calcium and magnesium), 2 mg/mL of acetaldehyde (Sigma-Aldrich, 00070) dissolved in PBS-, or 0.01 mg/mL of methylglyoxal (Sigma-Aldrich, M0252) dissolved in PBS-, were loaded into the nebulizer to generate an aerosol without heating the solutions. Both chemicals were soluble in PBS- and additional solvents were not needed. The solutions in the nebulizer were within concentration ranges reported in the literature for both aldehydes (Supplementary Tables S1-S2). Once the solutions were aerosolized, they were diluted by the air in the cloud chamber and only a small fraction of aerosol came in contact with each insert. In our prior studies with WS-23 (Wong et al., 2023), we determined the dilution factor in the cloud chamber to be about 150x.

Tissues were exposed to one puff of either PBS- control aerosol or aldehyde-containing aerosols, returned to the incubator for 4 h, then exposed to a second puff of PBS- or aldehyde-containing aerosols, after which they recovered in the incubator for 24 h before being processed for proteomics. Our exposure design (two puffs with a 4-h gap and then a 24-h recovery before lysing for proteomics) was based on previously published ALI studies in our lab using a VitroCell™ system (Nair et al., 2020; Wong et al., 2023). We opted to use only 2 puffs with a 4-h gap to recapitulate acute brief exposures that resemble those that an EC user would receive. The 24-h gap after the second puff was included to give cells time to translate proteins and is a standard recovery period for protein-based assays including proteomics, ELISAs, immunocytochemistry, and Western blots. Our experiments were done in triplicate with EpiAirway tissues from one donor.

### 2.3 TMT proteomics sample preparation

Protein concentrations in lysed EpiAirway™ tissue samples were determined using Pierce™ Protein Assay Kit (Thermo Scientific™, catalog number 23225). The volume of the lysed tissue samples was then adjusted to 500 µL in 50 mM of triethylammonium bicarbonate (TEAB, Sigma Aldrich, St. Louis, MO). The sample protein was precipitated in an equal volume of trichloroacetic acid and incubated on ice for 1 h. The supernatant was discarded, and proteins were rinsed with two subsequent 500 µL volumes of cold acetone, which were also discarded. Residual acetone was removed by SpeedVac. The dried pellets were resuspended in 50 µL of 50 mM TEAB then reduced with the addition of 1 μL of 500 mM tris(2-carboxyethyl)phosphine (Thermo Scientific, Rockford, IL) and incubated at 37 °C for 1 h. Next, 3 µL of 500 mM iodoacetamide (Sigma Aldrich) were added to each sample. Samples were incubated in the dark at room temperature for 1 h. Then 500 µL of 50 mM TEAB were added to the samples, along with trypsin/lysC mix (Promega, Madison, WI) at a 1:80 ratio to protein mass. Samples were digested at 37 °C overnight (16 h). The resulting peptide solution was purified with Waters HLB solid-phase extraction columns.

Sample peptide quantities were measured using a colorimetric peptide assay (Thermo Scientific). Samples were grouped into five separate TMT 10-plex runs. 3 μg was aliquoted from each sample for 10-plex Tandem Mass Tag labelling (Thermo Scientific, United States). Samples were adjusted to pH 8 using 1M TEAB, then reacted with a 1:1 mass ratio of reagent to peptide at room temperature for 1 h, before quenching with 8 µL of 5% hydroxylamine for 15 min. The labeled aliquots were combined into one sample, which was dried under SpeedVac in preparation for high-pH fractionation. The sample was resuspended in 0.1% trifluoroacetic acid, then fractionated into eight fractions using the Pierce high-pH fractionation kit (Thermo Scientific, United States). Individual fractions were dried under SpeedVac, then resuspended in 20 µL 0.1% formic acid for injection of the LC-MS system.

### 2.4 TMT proteomics analysis

Liquid chromatography was performed on a Thermo nLC1200 in single-pump trapping mode with a Thermo PepMap RSLC C18 EASY-spray column (2 μm, 100 Å, 75 μm × 25 cm) and a Pepmap C18 trap column (3 μm, 100 Å, 75 μm × 20 mm). Solvents were (A) water with 0.1% formic acid and (B) 80% acetonitrile with 0.1% formic acid. Samples were separated at 300 nL/min with a 130-min gradient starting at 3% B increasing to 30% B from 1 to 110 min, then to 85% B at 120 min and held for 10 min.

Mass spectrometry data were acquired on a Thermo Orbitrap Fusion in data-dependent mode. A full scan was conducted using 60k resolution in the Orbitrap in positive mode. Precursors for MS^2^ were filtered by monoisotopic peak determination for peptides, intensity threshold 5.0e^3^, charge state 2–7, and 60 s dynamic exclusion after one analysis with a mass tolerance of 10 ppm. Collisional-induced dissociation spectra were collected in ion trap MS^2^ at 35% energy and isolation window 1.6 m/z. Synchronous Precursor Selection MS^3^ was utilized for TMT ratio determination at HCD energy 65%.

Results were searched individually in Proteome Discoverer 2.2 (Thermo Scientific) against the UniProt FASTA database for *Homo sapiens* UP000005640. The precursor mass tolerance was set to 10 ppm and fragment mass tolerance to 0.6 Da. Fixed modifications were carbamidomethyl (Cys +57.021 Da), TMT 6plex (Lys, N-terminus +229.163 Da), and dynamic modifications included methionine oxidation (+15.995 Da) N-terminal acetylation (+42.011 Da). Results were filtered to a strict 1% false discovery rate. Abundances from reporter ions in MS^3^ were normalized to total peptide amount and scaled according to normalization of the control sample between runs. Ratios were generated and their associated p-values were calculated using individual protein ANOVA considering biological replicates. The exposure groups were compared to the control group (PBS treated), and differentially expressed proteins (DEPs) were considered significant using a cut-off of an adj p-value <0.05. The full proteomics data file is provided as an excel file (Supplementary Table S3).

### 2.5 IPA analysis

The full proteomics spreadsheet for the acetaldehyde vs. PBS and methylglyoxal vs. PBS were uploaded into Ingenuity Pathway Analysis software (IPA) (Qiagen Inc., Germantown, MD. USA), and unnamed proteins were identified using Uniprot ID matching (https://www.uniprot.org/id-mapping). An expression analysis was run using proteins with an adj p < 0.05. For all IPA analyses, default settings and cutoffs were used. Default settings for IPA the Tox List was -log_2_fold >1.3. Circle plots were generated using circlize in R to visualize relationships of proteins to specific tox functions provided in the tox list.

### 2.6 Gene ontology (GO) analysis

Protein lists for different exposure groups were uploaded to the Gene Ontology website, significant proteins were selected using a cutoff of adj p-value <0.05. GO Terms from the three ontologies; Biological Process, Cellular Compartment, and Molecular Function, were extracted clustered using the rrvgo package in R (Sayols, 2025). Similar GO Terms are grouped, and one is designated as a “parent term”, to describe the entire group. Circle plots were also created to visualize relationships of proteins to different GO Terms. A heatmap was generated with a distance matrix to cluster similar proteins based off GO Term overlap. GO Term overlap was calculated with the GOSemSim package on R (Yu et al., 2010).

### 2.7 BEAS-2B cell culture

Human bronchial epithelial cells (BEAS-2B cells) were used for endpoint assays to further support the proteomics data. Cells were obtained from the American Type Culture Collection (ATCC, Manassas, Virginia; Cat No CRL-3588) and cultured in BEBM^®^ (bronchial epithelial cell growth basal medium) with BEGM^®^ (bronchial epithelial cell growth medium SingleQuots^®^ supplements and growth factors) from Lonza (Catalog #: CC-3171 and CC-4175), using a method described previously in detail (Nair et al., 2020; Omaiye et al., 2019). Nunc T-25 tissue culture flasks were coated for 2 h with BEBM, collagen, bovine serum albumin, and fibronectin prior to subculturing. Upon reaching 90% confluency, cells were rinsed with Dulbecco’s phosphate-buffered saline (DPBS) without calcium and magnesium then detached using 1.5 mL of 0.25% trypsin EDTA/DPBS with polyvinylpyrrolidone for 1.5 min at 37 °C. Cells were passaged into T-25 flasks at 75,000 cells/flask, and the medium was changed every other day. For all assays except phalloidin, BEAS-2B cells were plated at a density of ∼21,000 cells/cm^2^ and allowed to attach and expand for 24 h, then treated in cell culture plates for different lengths of time depending on the assay. Concentrations used for each experiment were optimized for each chemical and for each assay. Methylglyoxal caused cells to die/detach at lower concentrations than acetaldehyde and was used at lower concentrations across all assays.

### 2.8 MTT assay

MTT (3-(4,5-dimethylthiazol-2-yl)-2,5-diphenyltetrazolium bromide) assays were performed as described in detail previously (Omaiye et al., 2019; Omaiye et al., 2024). BEAS-2B cells were plated in 96-well plates at a density of 7,000 cells/well. Cells were grown for 24 h, then treated with either acetaldehyde or methylglyoxal for 24 h. A stock solution of acetaldehyde (Sigma-Aldrich, 00070) was prepared at 1 mg/mL (22.7 mM) in culture medium, and a 3-fold serial dilution was performed across six concentrations. A stock solution of methylglyoxal (Sigma-Aldrich, M0252) was prepared at 0.1 mg/mL (1.37 mM) in culture medium, and a 3-fold serial dilution was likewise performed across six concentrations. Final concentrations for the aldehydes were similar (0.003 mg/mL, 0.001 mg/mL, 0.03 mg/mL, 0.1 mg/mL, 0.3 mg/mL, 1 mg/mL for acetaldehyde and 0.00003 mg/mL, 0.001 mg/mL, 0.003 mg/mL, 0.01 mg/mL, 0.03 mg/mL, 0.1 mg/mL for methylglyoxal). The high concentration for methylglyoxal was lower because 1 mg/mL produced a vapor effect in untreated control wells. Separate 96-well plates were used for each chemical exposure and its controls to avoid vapor effects (Behar et al., 2012). An additional plate containing untreated controls in BEGM was also included. MTT reagent was added after 24 h of exposure (48 h after plating). Formazan crystals were solubilized using 99.5% dimethyl sulfoxide (DMSO), and the absorbance was read at 570 nm using a Bio-Tek Synergy HTX plate reader (Agilent, Santa Clara, CA, United States). For each variable tested, three independent experiments were performed. MTT assays were analyzed using a one-way ANOVA with Dunnett’s multiple comparisons post-hoc test. The IC_50_ of the methylglyoxal exposure was determined by nonlinear regression using an inhibitor versus response variable slope four parameters analysis. The IC_50_ of the acetaldehyde exposure was determined by nonlinear regression using an inhibitor versus normalized response variable slope analysis. IC_70_ of both graphs was determined with the formula: IC70=70100‐701H×IC50 , which uses the Hill Slope value (H) and the IC_50_ results of the nonlinear regression results. The ${\text{IC}}_{70}$ is used according to section B.2.3.7 of the ISO protocol 10993–5 (International Organization for Standardization, 2009) to determine if the exposure is potentially cytotoxic.

TRPA1 (Torcis Bioscience, AM 0902) and TRPM8 (Torcis Bioscience, TC-I 2014) antagonists were used to examine the contribution of TRP channels to changes in mitochondrial reductases. The antagonists were shipped in powder form and resuspended in DMSO, as recommended by Torcis Bioscience. Serial dilutions were made from the stock solution. The highest concentrations of DMSO were 1.5 × 10^−6^% for TRPA1 and 1.9 × 10^−6^% for TRPM8. These concentrations did not produce any effect in the MTT. Using the same plating and MTT assay protocol, BEAS-2B were treated with the MTT IC_50_ concentration (acetaldehyde = 8260 µM and methylglyoxal = 262 µM) in the presence or absence of the TRPA1 and TRPM8 antagonists. TRPA1 and TRPM8 antagonists were used over a range of 10^−8^ μM–10^−3^ μM, while the concentration of the test aldehyde remained fixed. Cells were first pretreated with the TRPA1 or TRPM8 antagonists for 15 min and then treated with both the chemical and antagonist for 24 h.

### 2.9 CellRox

Reactive oxygen species were visualized in BEAS-2B cells using a method described previously (Khachatoorian et al., 2021). Cells were plated in ibidi μ-Slide 8-well chamber slides (Ibidi, Fitchburg, WI) at a density of 10,000 cells/cm^2^. Following a 24-h attachment period, cells were incubated with acetaldehyde (Sigma-Aldrich, 00070) at final concentrations of 0.01 mg/mL (0.0227 mM), 0.1 mg/mL (0.227 mM) and 1 mg/mL (2.27 mM) or methylglyoxal (Sigma-Aldrich, M0252) at final concentrations of 0.0001 mg/mL (0.00139 mM), 0.001 mg/mL (0.0139 mM), and 0.01 mg/mL (0.139 mM) for 24 h. Non-treated control wells (0 mg/mL of chemical) contained BEGM (culture medium) only. Our ranges were selected to capture non-cytotoxic and cytotoxic concentrations. The cells were washed three times with PBS+ (Dulbecco’s Phosphate Buffered Saline; Gibco/Thermo Fisher, Waltham, MA) then incubated in 1 μM CellROX^®^ Green staining solution (Invitrogen, Carlsbad, CA) for 30 min. Cells were rewashed, then stained using Vectashield with DAPI mounting medium (Vector Laboratories, Burlingame, CA). Immediately after, cells were imaged using a ×60 water immersion objective on a Nikon Eclipse Ti-E inverted microscope (Nikon Instrument, Melville, NY, USA); Images were captured with a high-resolution Andor Zyla VSC-04941 camera (Andor, Belfast, UK).

### 2.10 Phalloidin labeling

TRPA1 (Torcis Bioscience, AM 0902) and TRPM8 (Torcis Bioscience, TC-I 2014) antagonists were also used to examine the contribution of TRP channels to changes in the actin cytoskeleton. BEAS-2B cells were plated in ibidi 8-well chamber slides at 10,000 cells/well. After 48 h, cells were pretreated for 15 min with either no antagonist, TRPA1 antagonist, or TRPM8 antagonist. TRPA1 and TRPM8 antagonists were both used at a concentration of 10^−6^ μM. After preexposure, we removed half the medium from each well and added an equivalent volume of test chemical and treated for 24 h with acetaldehyde or methylglyoxal. Final acetaldehyde concentrations ranged from 0.05 mg/mL to 0.5 mg/mL, while final methylglyoxal concentrations ranged from 0.0026 mg/mL to 0.026 mg/mL. After exposure, cells were fixed with 4% paraformaldehyde for 10 min, then stained with 1x Phalloidin-iFluor 594 ab176757 (Abcam, Cambridge, United Kingdom) for 1 h, following the assay procedure recommended by the manufacturer. Cells were labeled with DAPI and imaged at 60x with a Nikon Eclipse Ti inverted microscope equipped with an Andor Zyla VSC-04941 camera.

## 3 Results

### 3.1 Acetaldehyde and methylglyoxal altered the proteome of EpiAirway™ tissues

**FIGURE 1:**
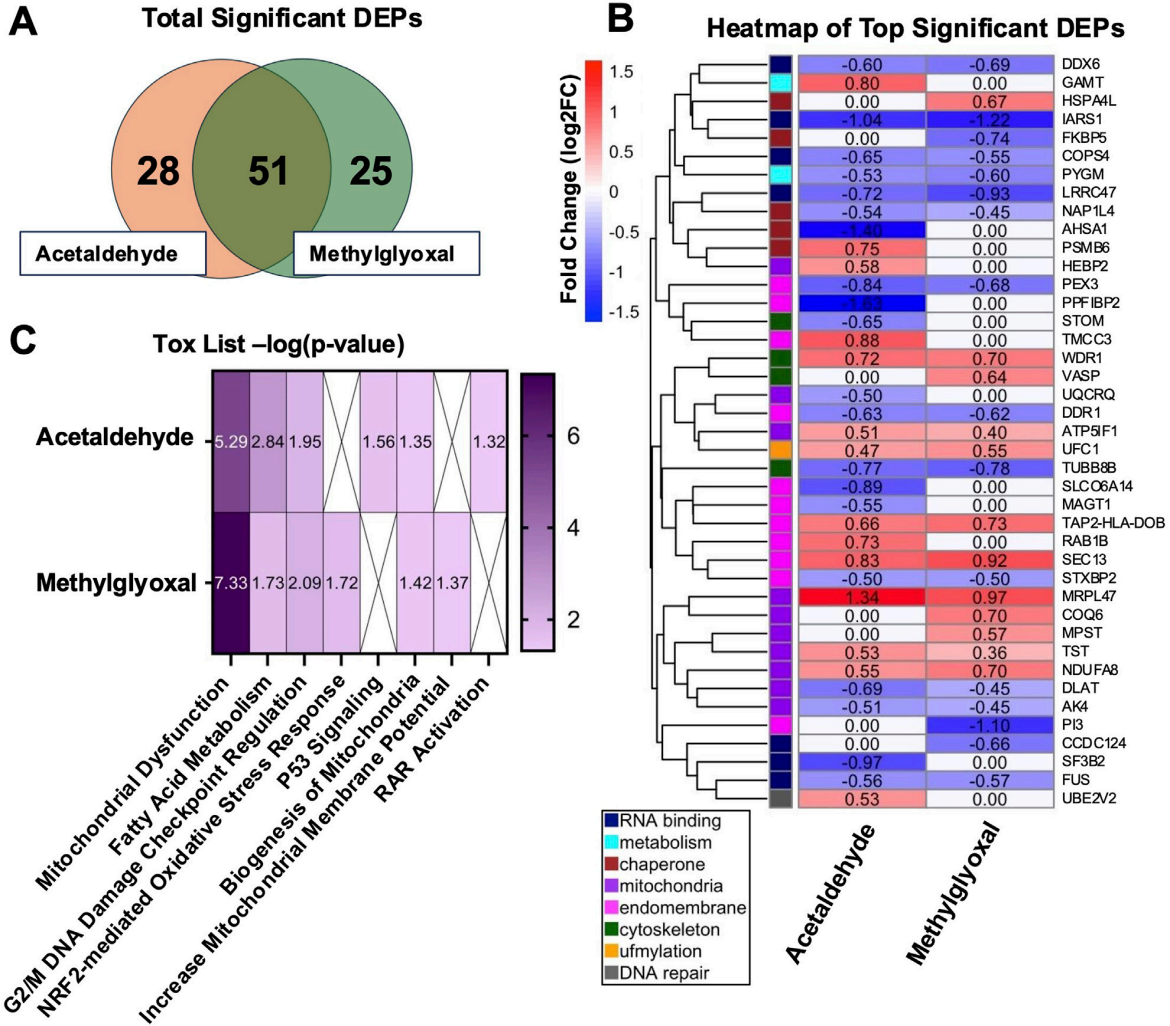
DEPs in EpiAirway™ treated with acetaldehyde or methylglyoxal and IPA Tox List. **(A)** Venn diagram of total significant DEPs (adj p < 0.05). **(B)** Heat map of top significant DEPs, clustered by similar GO Terms and categorized by color based on function. **(C)** Heatmap of IPA Tox List Analysis showing the significance (-log p-value) of each toxicological function for both chemicals.

The purpose of our proteomics experiment was to obtain an overview of the processes and proteins that were changing following acute exposure to the two aldehydes at realistic exposure concentrations. It was expected that the proteomics data would provide insight into the major targets of the aldehydes, and these would be followed up in concentration-response experiments using submerged cultures with BEAS-2B cells. Following exposure of EpiAirway™ tissues at the ALI to either acetaldehyde or methylglyoxal, proteomics analysis identified 79 differentially expressed proteins (DEPs) in the acetaldehyde group and 76 DEPs in the methylglyoxal group (Figure 1A). 51 DEPs overlapped in the two exposure groups, with a 65% overlap in acetaldehyde and a 67% overlap in methylglyoxal. Significant DEPs were determined with an adjusted p-value <0.05.

Top DEPs were plotted in a heatmap and clustered using a distance matrix based on GO Term relationships (Figure 1B). The relatively low aldehyde concentrations and short duration of the exposures produced modest fold changes in the DEPs. After clustering, eight categories related to cell processes were identified and are shown using a color scale based on hierarchical clustering (Figure 1B). Acute exposures to low concentrations of these aldehydes affected a broad range of cellular processes that included RNA binding, metabolism, chaperone, mitochondria, endomembrane, cytoskeleton, ufmylation, and DNA repair. When DEPs overlapped in the two exposures, their fold changes were very similar, supporting the idea that acetaldehyde and methylglyoxal produce similar responses in cells. For example, five of the seven DEPs involved in RNA binding and RNA processing were downregulated in both groups. Likewise, ufmylation, a post-translational modification that attaches a ubiquitin-fold modifier, was upregulated in both the acetaldehyde and methylglyoxal groups.

### 3.2 IPA tox list analysis

**FIGURE 2:**
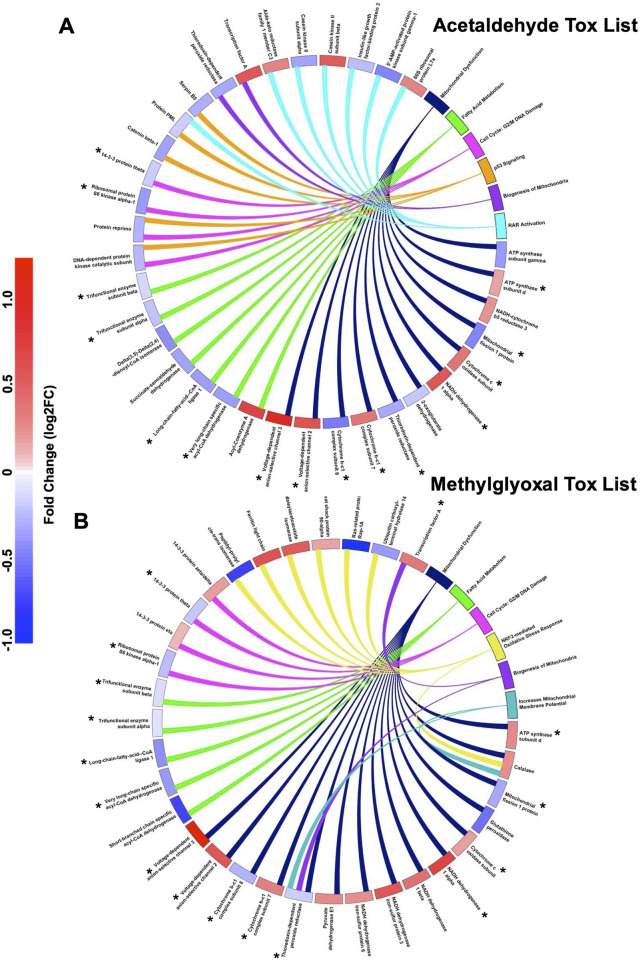
IPA Tox lists for DEPs for both chemicals. Circle plots show the log_2_ fold change for proteins involved in IPA toxicological functions for acetaldehyde **(A)** and methylglyoxal **(B)**. The colored scale bar shows the magnitude of the fold change for each DEP in the circle plot with blue indicating downregulation and red indicating upregulation. The toxicological functions have colors other than blue and red and are connected by lines to proteins within their functions. Toxicological functions that overlap in both chemicals are colored the same between the two circle plots.

IPA Tox List Analysis identified the top seven toxicological functions affected by acetaldehyde and methylglyoxal, four of which were shared by both chemicals (Figure 1C). Circle plots show proteins mapped to respective Tox List functions in acetaldehyde (Figure 2A) and methylglyoxal (Figure 2B) treated EpiAirway™ tissue. The overlapping toxicological functions in the two exposure groups were Mitochondrial Dysfunction, Fatty Acid Metabolism, Cell Cycle: G2/M DNA Damage Checkpoint Regulation, and Biogenesis of Mitochondria. For Mitochondrial Dysfunction, there were 11 DEPs in acetaldehyde exposures, 15 DEPs in methylglyoxal, and 9 which overlapped in both exposures. For Fatty Acid Metabolism, there were 7 DEPs from acetaldehyde and 5 DEPs from methylglyoxal, 4 of which overlapped between both exposures. For G2/M DNA Damage Checkpoint Regulation, there were 4 DEPs from acetaldehyde and 4 DEPs from methylglyoxal, with 2 overlapping between exposures. For Biogenesis of Mitochondria, there were 2 DEPs that were the same for both acetaldehyde and methylglyoxal. In all cases, overlapping proteins (indicated by asterisks) in the two exposure groups had similar direction of change and fold changes (Figures 2A,B).

Unique Tox List hits for acetaldehyde treated EpiAirway™ included p53 signaling and RAR activation (Figure 2A). Unique Tox Lists hits for methylglyoxal included NRF2-mediated Oxidative Stress Response and Increases Mitochondrial Membrane Potential (Figure 2B).

### 3.3 GO analysis identified overlapping processes affected by exposures

**FIGURE 3:**
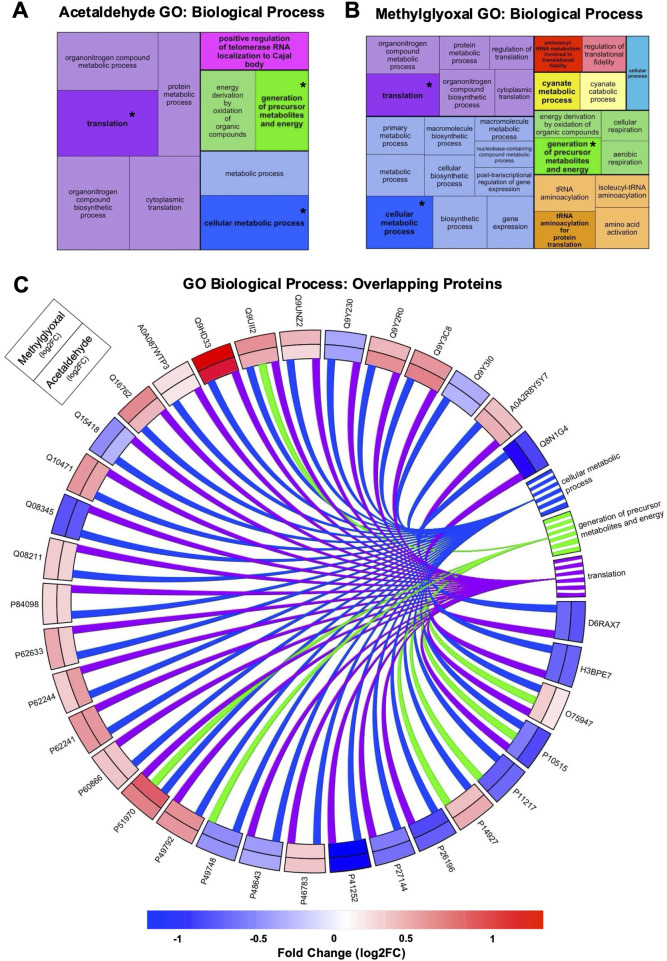
GO Terms for Biological Processes from acetaldehyde and methylglyoxal treated EpiAirway™. Treemaps of GO Biological Processes for acetaldehyde **(A)** and methylglyoxal **(B)** treated EpiAirway™. GO Terms were clustered based on similarity using rrvgo. In each cluster the parent GO Term is bolded and darker than other terms in the cluster. Box size corresponds to significance (–log10 (p-value)) with more significant terms having larger boxes. Treemaps are not scaled across **(A)** and **(B)**. Overlapping GO Terms between the two chemicals are colored alike and asterisks indicate parent GO Terms found in both groups. **(C)** GO Biological Processes overlapping in the acetaldehyde and methylglyoxal groups. A total of 35 proteins overlapped in the two chemical exposures which corresponded to three common biological processes. Log 2-fold changes displayed on the outer track are for methylglyoxal and the inner track for acetaldehyde. The color scale indicates the magnitude of the fold change for different proteins.

**FIGURE 4:**
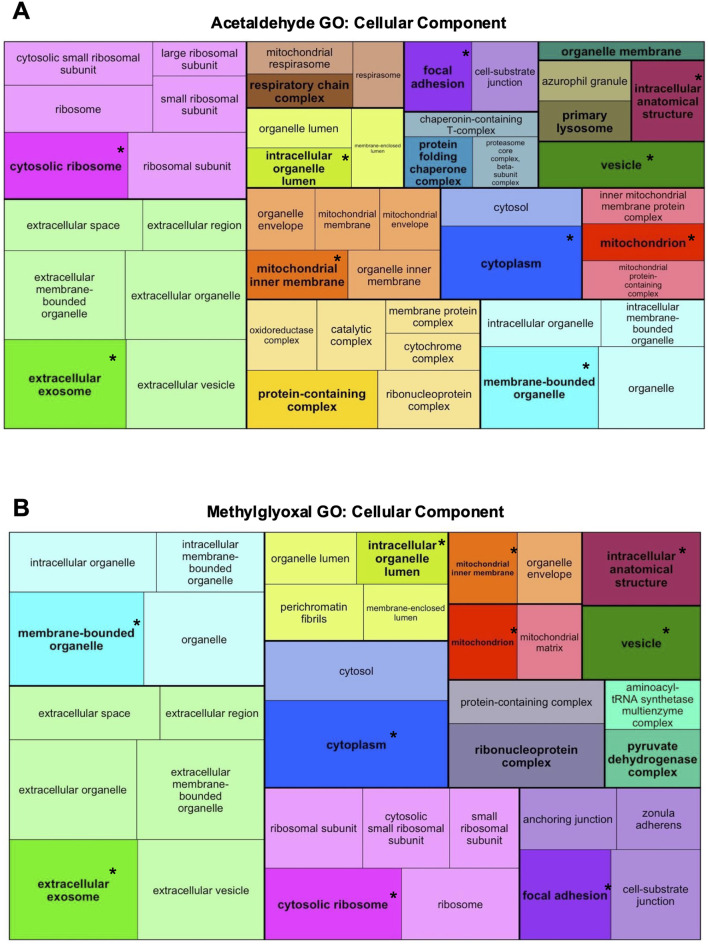
Acetaldehyde and methylglyoxal treated GO Terms for Cellular Component ontology. All the DEPS were used to generate treemaps of GO Cellular Component for acetaldehyde **(A)** and methylglyoxal **(B)** groups. GO Terms were clustered based on similarity using rrvgo. Each cluster has one parent GO Term that is bolded and darker than other terms in the cluster. Within individual treemaps, box size corresponds to significance (–log10 (p-value)) with more significant terms having larger boxes. Treemaps are not scaled across **(A,B)**. Overlapping GO Terms between the two groups are colored alike and asterisks indicate parent GO Terms found in both groups.

GO Analysis was used to identify GO Terms for the three ontologies, Biological Process (Figure 3), Molecular Function (Supplementary Figure S1), and Cellular Component (Figure 4). GO Terms for each ontology were clustered based on similarity using the R package rrvgo, and one GO Term from each category is designated as the “parent term” (GO Term with highest -log10 adj p-value). Parent GO Terms are bolded and a darker shade than the rest of the GO Terms in that group, with overlapping terms indicated by an asterisk. GO Terms for all three ontologies had considerable overlap between the two exposures. GO Biological Processes include broad scale processes that can encompass a series of events or activities that involve multiple gene products. GO Molecular Function describes a specific biochemical function of individual gene products at the molecular level. GO Cellular Component describes the cellular location of the gene products.

#### 3.3.1 GO biological processes

In GO Biological Processes ontology, acetaldehyde and methylglyoxal treated EpiAirway™ overlapped in three parent GO Terms: translation, generation of precursor metabolites and energy, and cellular metabolic process (Figures 3A,B). Overlapping proteins from both groups were mapped to GO Biological Process parent terms in a circle plot (Figure 3C). Thirty-five proteins overlap in the two exposures for these GO Terms. For each protein, both the direction of fold change and the magnitude of the fold change were similar between the two exposures (Figure 3C). Four Biological GO Terms were found only in the methylglyoxal group (Supplementary Figure S2), while there was one unique Biological GO Term in the acetaldehyde group.

#### 3.3.2 GO molecular function

GO Molecular Function had fewer total GO Terms for acetaldehyde and methylglyoxal, with only two overlapping parent GO Terms: RNA binding and structural constituent of ribosome (Supplementary Figure 1A, B). For methylglyoxal, there were six unique GO Terms (Supplementary Figure S1C).

#### 3.3.3 GO cellular component

**FIGURE 5:**
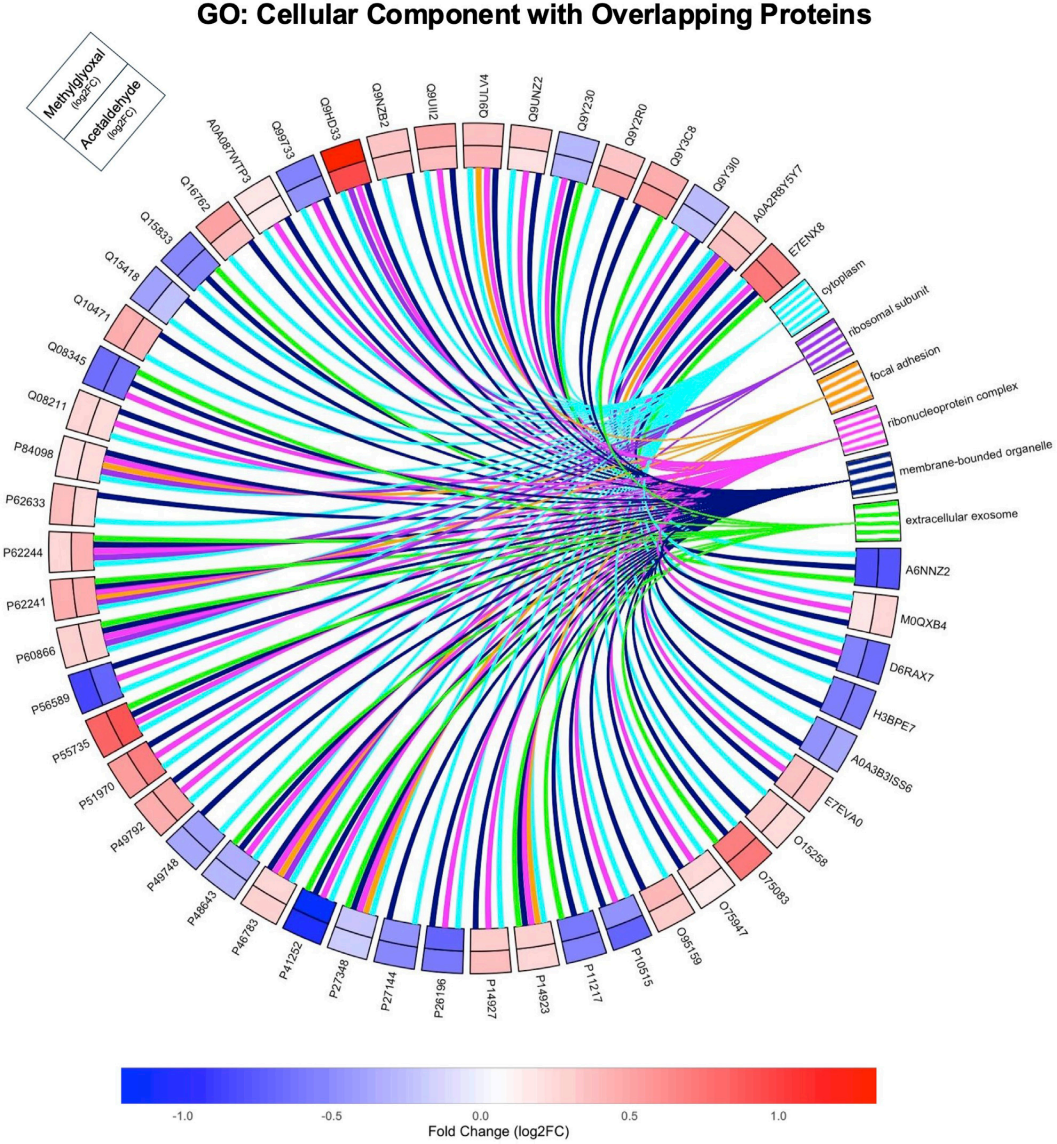
GO Cellular Component Overlapping Pathways. Circle plot showing the relationship of the 51 overlapping DEPs from both groups to overlapping GO Terms from the Cellular Component ontology. The GO analysis for this figure was run with the 51 overlapping proteins only. Log 2-fold changes on the outer track are for methylglyoxal while the inner track is for acetaldehyde. The color scale indicates the magnitude of the fold change for different proteins.

In GO Cellular Component ontology, most of the Parent GO Terms overlapped between the acetaldehyde (10/15 Parent GO Terms) and methylglyoxal (10/12 Parent GO Terms) treated groups. Overlapping Parent GO Terms included: extracellular exosome, focal adhesion, cytosolic ribosome, cytoplasm, vesicle, intracellular anatomical structure, membrane bound organelle, intracellular organelle lumen, mitochondrion, and mitochondrial inner membrane (Figures 4A,B). Overlapping GO Terms were mapped to their proteins in a circle plot (Figure 5). Remarkably, all of the protein pairs were altered in the same direction and had similar log_2_ fold changes.

### 3.4 Effects of acetaldehyde and methylglyoxal on BEAS-2B Cells grown in submerged culture

Concentration-response experiments were next done using BEAS-2B cells grown in submerged culture to confirm the major effects discovered in the proteomics data and to further investigate the relative potency of each aldehyde. The processes in these follow-up experiments (mitochondrial dysfunction, oxidative stress, and cytoskeletal depolymerization) were chosen as they were major targets of the chemical exposures in the proteomics analysis.

#### 3.4.1 Effect of acetaldehyde and methylglyoxal on mitochondria and cellular respiration

**FIGURE 6:**
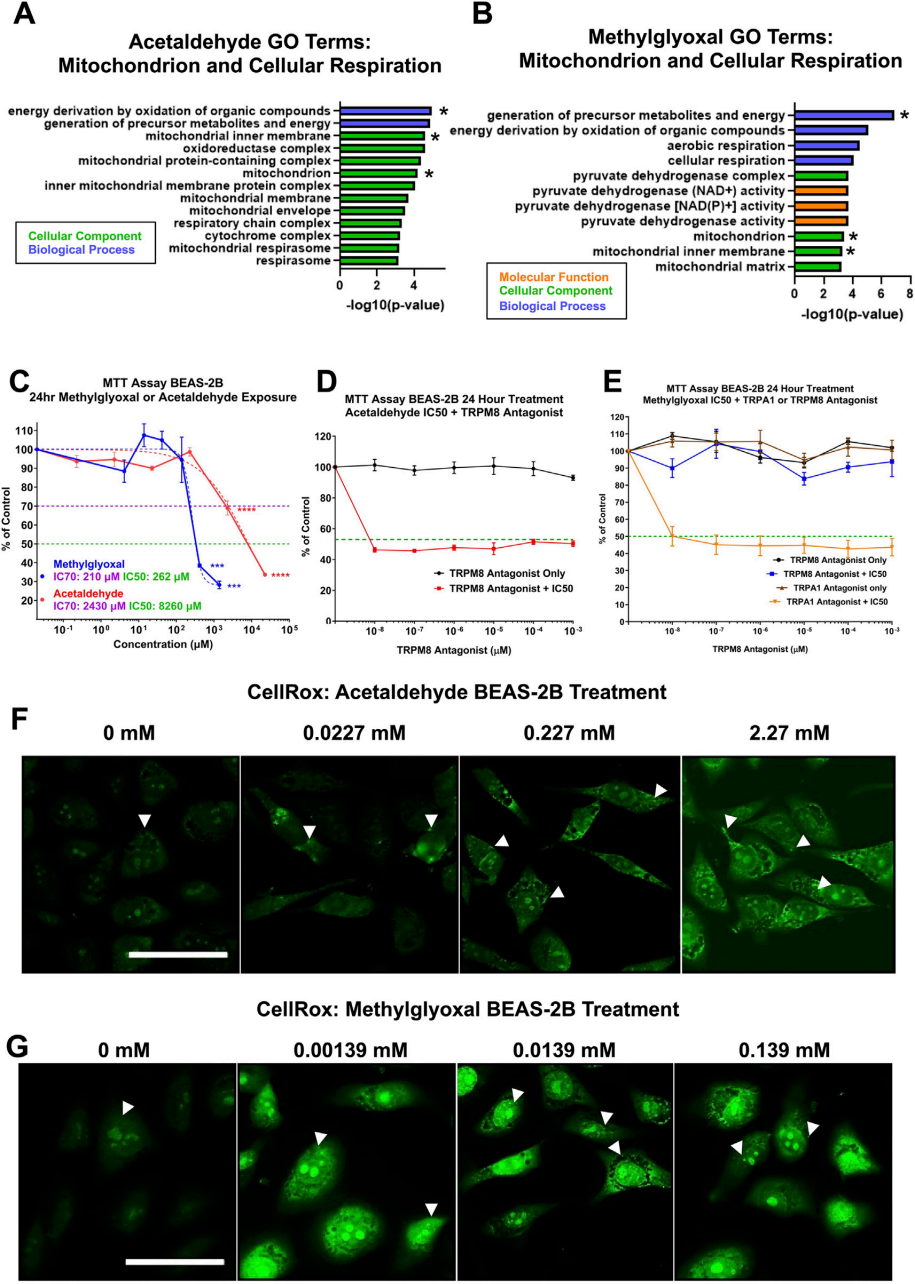
Cytotoxicity of acetaldehyde and methylglyoxal in mitochondrial assays and involvement of the TRPM8 channel. **(A,B)** GO Terms related to mitochondria, cellular respiration, and oxidative phosphorylation for acetaldehyde and methylglyoxal, overlapping GO Terms are indicated with an asterisk. **(C)** MTT assays were performed on monolayers of BEAS-2B cells in submerged culture for acetaldehyde and methylglyoxal. One-way ANOVA with Dunnett’s multiple comparisons post-hoc test was used to evaluate significance, p < 0.001 (***) and p < 0.0001 (****). **(D)** The TRPM8 channel antagonist did not prevent the effect of acetaldehyde in the MTT assay. **(E)** The TRPM8 channel antagonist, but not the TRPA1 channel antagonist, prevented methylglyoxal from decreasing mitochondrial reductase activity. In C-E, the green dashed line shows 50% inhibition vs. the control. In D and E, the IC_50_ concentration for acetaldehyde and methylglyoxal would be expected to fall on this line if the antagonist produced no effect. **(F,G)** Acetaldehyde and methylglyoxal increased mitochondrial and nuclear ROS in the CellROX™ assay (white arrows).

GO Terms related to mitochondria, cellular respiration, and oxidative stress were extracted and plotted onto bar graphs for both acetaldehyde and methylglyoxal; overlapping GO Terms are indicated with an asterisk (Figures 6A,B). Both exposure groups had two overlapping GO Terms from the Biological Processes ontology: (1) energy derivation by oxidation of organic compounds, and (2) generation of precursor metabolites and energy. Additionally, methylglyoxal also included cellular respiration and aerobic respiration (Figure 6A). Acetaldehyde produced 11 GO Terms from the Cellular Component ontology, whereas methylglyoxal produced four. Acetaldehyde yielded no GO Terms from the Molecular Function ontology related to the mitochondria, while methylglyoxal yielded three (Figure 6B).

#### 3.4.2 Effect of acetaldehyde and methylglyoxal on mitochondrial reductases

Some of the major processes affected in the proteomics analysis were studied further *in vitro* using BEAS-2B cells in submerged culture. Since mitochondrial dysfunction was a major effect observed in both acetaldehyde and methylglyoxal Tox Lists, MTT assays were run to determine how mitochondrial reductases performed during exposures. While both acetaldehyde and methylglyoxal produced cytotoxicity in the MTT assay, methylglyoxal was effective at a much lower concentration (IC_50_ for acetaldehyde = 8260 µM and IC_50_ for methylglyoxal = 262 µM) (Figure 6C). When acetaldehyde was tested in the MTT assay at its IC_50_ concentration, its effect was not reversed by the TRPM8 antagonist (Figure 6D). However, when methylglyoxal was tested at its IC_50_ concentration, its effect was completely reversed by 10^−6^ μM of the TRPM8 antagonist (TC-I 2014) (Figure 6E). TRPA1 antagonist did not rescue the effect of methylglyoxal in the MTT assay. The antagonists alone produced no effect in the MTT assay (Figures 6D,E).

#### 3.4.3 Acetaldehyde and methylglyoxal induce oxidative stress in BEAS-2B Cells

CellRox, a probe that is oxidized in the presence of reactive oxygen species (ROS), was used to evaluate oxidative stress in treated cells. In both acetaldehyde and methylglyoxal treated BEAS-2B cells, CellRox signal increased in a dose-dependent manner for both chemicals. Fluorescent signal was mainly in the mitochondria in the acetaldehyde treated group, while both the mitochondria and nucleoli were labeled in the methylglyoxal group (Figures 6F,G) Fluorescent intensity was greater in methylglyoxal than in acetaldehyde.

#### 3.4.4 Actin cytoskeleton depolymerization and adhesion inhibition by acetaldehyde and methylglyoxal

**FIGURE 7:**
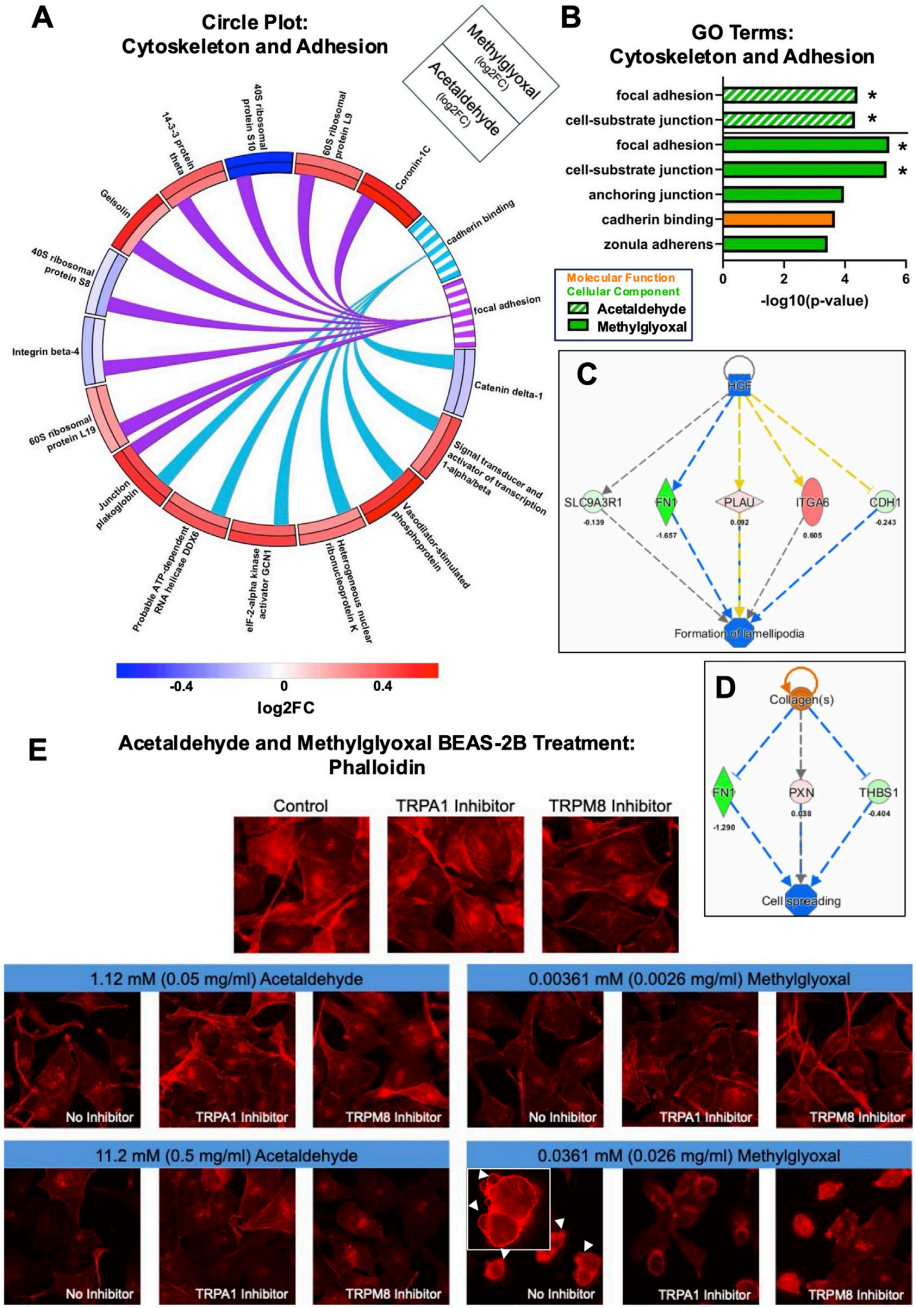
Acetaldehyde and methylglyoxal affected the actin cytoskeleton. **(A)** Circle plot connecting proteins to GO Terms related to actin cytoskeleton. Log 2-fold changes in the outer and inner track are for methylglyoxal and acetaldehyde, respectively. The color scale shows the magnitude of the fold change for each protein. **(B)** GO Terms related to the actin cytoskeleton and cell adhesion for acetaldehyde and methylglyoxal, overlapping GO Terms are indicated with an asterisk. IPA regulator effects for acetaldehyde **(C)** and methylglyoxal **(D)** treated EpiAirway™ predicted inhibition of cell spreading and lamellipodia formation. **(E)** Exposure of BEAS-2B monolayers caused depolymerization of phalloidin-labeled actin, a decrease in cell-cell adhesion, and cell rounding. Methylglyoxal at 0.0361 mM induced dynamic blebbing (white arrows), which was partially prevented by the TRPA1 and TRPM8 antagonists.

Acetaldehyde and methylglyoxal produced GO Terms related to cell adhesion (focal adhesion and cadherin binding), and both fold change and direction of change were similar for each pair of affected proteins (Figure 7A). GO Terms for acetaldehyde and methylglyoxal related to cytoskeleton and adhesion were extracted and plotted in a bar graph (Figure 7B). In the proteomics analysis, both acetaldehyde and methylglyoxal were predicted to decrease polymerization of f-actin and impair cell processes dependent on actin (e.g., acetaldehyde decreased cell spreading and methylglyoxal decreased formation of lamellipodia) (Figures 7C,D). BEAS-2B cells were labeled with phalloidin, a fungal peptide conjugated with a fluorochrome, that binds to f-actin (Figure 7E). Acetaldehyde (1.12 mM) and methylglyoxal (0.0036 mM) caused a decrease in phalloidin intensity, indicating a decrease in polymerized actin filaments. In higher concentrations, acetaldehyde (11.2 mM) further decreased phalloidin fluorescence, which was partially rescued by addition of a TRPA1 (AM 0902) or TRPM8 antagonist (TC-I 2014). In our high concentration of methylglyoxal (0.0361 mM), actin depolymerized, and cells were rounded and exhibited dynamic blebbing. Addition of the TRPA1 or TRPM8 antagonists prevented dynamic blebbing, but the cells were still rounder and less spread than the controls (Figure 7E).

## 4 Discussion

The global effects of acetaldehyde and methylglyoxal exposure on EpiAirway™ tissues were compared using proteomics analysis following two brief exposures at concentrations that were within the ranges reported in EC aerosols. Both chemicals produced significant changes in protein expression with DEPs identified by an adjusted p-value <0.05. These DEPs were associated with toxicological outcomes and perturbations in biological, cellular, and molecular processes. Notably, there was considerable overlap in the identity and directionality of fold change for DEPs between the two chemicals. Key toxicological pathways impacted included mitochondrial dysfunction, altered fatty acid metabolism, and G2/M cell cycle DNA damage checkpoints. Circle plots of Gene Ontology (GO): Cellular Component revealed striking overlap in affected components, with both aldehydes exerting negative effects on mitochondria and the actin cytoskeleton. These proteomic findings were supported by concentration–response experiments using submerged BEAS-2B cells, which demonstrated mitochondrial dysfunction, elevated oxidative stress, reduced f-actin stability, and impaired cell spreading. Importantly, methylglyoxal elicited these effects at concentrations approximately 200-fold lower than those required for acetaldehyde, in both the ALI proteomics and BEAS-2B assays.

Both chemical had similar proteomics effects in our ALI exposures, even though the tested amounts in the nebulizer-generated aerosols were 200x lower for methylglyoxal (2 µg/puff) than acetaldehyde (400 µg/puff). Acetaldehyde concentrations in published EC aerosols range from 7.5 × 10^−8^ μg/mL to 72 μg/mL across 48 studies [Supplementary Table S1] and for methylglyoxal range from 1.7 × 10^−4^ μg/mL to 0.82 μg/mL across 16 studies [Supplementary Table S2]. These ranges are broad and are affected by many factors, such as wattage, flavor chemicals, coil metal, wick material, puff topography, trapping method, and chemistry of the EC fluid (Jensen et al., 2017; Uchiyama et al., 2020; Bekki et al., 2014; Son et al., 2020; Li et al., 2020; Kośmider et al., 2018; Saliba et al., 2018; Qu et al., 2018). After accounting for the dilution factor in the VitroCell™ exposure system (∼150-fold), the concentrations used in our study fall within the range that users might receive (e.g., estimated acetaldehyde concentration = 5.3 ug/mL and estimated methylglyoxal concentration = 0.026 ug/mL from two puffs).

Aerosols in our study were generated using a vibrating mesh nebulizer within a cloud chamber, to minimize thermal degradation and the formation of confounding reaction products associated with coil-based ECs. Our exposure platform is particularly relevant to a new class of ECs that employ ultrasonic aerosolization, such as SURGE™ (Omaiye et al., 2024; Omaiye and Talbot, 2025). Lost Mary, a sub-brand of ELFBAR, now offers a high-puff-count ultrasonic disposable EC (Lost Mary Ultrasonic 35K Puffs). These emerging devices may generate aerosols with different chemical profiles, potentially including higher concentrations of methylglyoxal (MGO) not typically observed in standard coil-based ECs. As evidence of this, a recent analysis of SURGE™ ultrasonic ECs reported methylglyoxal concentrations of approximately 100 μg/mL - substantially higher than acetaldehyde, which was present at <10 μg/mL (Omaiye et al., 2024)

Although SURGE™ devices produce relatively less heat (∼132 °C) than traditional ECs, this is significantly lower than the temperatures generated by conventional metal coils. The thermal degradation of EC solvents, particularly propylene glycol, is well characterized: it undergoes oxidation to form methylglyoxal, which can subsequently be converted to acetaldehyde and formaldehyde. Saliba et al. (2015) demonstrated that in metal coils, methylglyoxal formation peaks at ∼256 °C, whereas acetaldehyde formation peaks at higher temperatures (∼360 °C) (Saliba et al., 2018). While most studies report acetaldehyde at higher concentrations than methylglyoxal in EC aerosols (Supplementary Tables S1, S2), devices operating at lower power levels can produce relatively more methylglyoxal (Supplementary Tables S1, S2) (Azimi et al., 2021; Omaiye et al., 2024). Collectively, these findings support the conclusion that methylglyoxal formation is favored over acetaldehyde under lower-temperature conditions, such as those present in ultrasonic or low-power EC devices.

Methylglyoxal was more potent than acetaldehyde in all assays conducted across both the ALI and submerged exposure platforms. Previous studies comparing the toxicity of diacetyl and methylglyoxal similarly reported greater toxicity of methylglyoxal at lower concentrations. In one such study, rats exposed to methylglyoxal or diacetyl exhibited significantly more necrosis in the nasal and bronchiolar epithelium following methylglyoxal exposure (Hubbs et al., 2019). Although acetaldehyde and diacetyl are well-recognized constituents of EC aerosols and are commonly studied for their role in EC-related toxicity, fewer studies have examined methylglyoxal in this context even though methylglyoxal is more toxic than both at lower concentrations. Our findings demonstrate that methylglyoxal is more potent than acetaldehyde at lower concentrations, highlighting the need for increased attention to this chemical, which may have been overlooked due to its relatively lower reported levels in EC emissions.

Multiple toxicological effects associated with acetaldehyde and methylglyoxal were observed in both EpiAirway™ tissues and cultured BEAS-2B cells. In the EpiAirway™ model, proteomic analysis identified disrupted pathways related to NRF2-mediated oxidative stress (IPA Toxicity List), mitochondrial function, and cellular respiration—findings consistent with previous reports in other cell types (Yan et al., 2023; Takeuchi et al., 2024; Kim et al., 2004; Ramasamy et al., 2006; Novitskiy et al., 2006; Desai et al., 2010; Tamura et al., 2014; Lee and Chang, 2014). Mitochondrial involvement suggested by proteomic data was corroborated by functional assays in BEAS-2B cells, where both chemicals significantly reduced mitochondrial reductase activity (MTT assay) and increased reactive oxygen species (ROS) levels in the mitochondria and nuclei (CellROX™ assay). Inhibition of TRPA1 did not rescue mitochondrial reductase activity; however, TRPM8 inhibition restored activity to control levels in methylglyoxal-treated cells, suggesting a specific interaction between methylglyoxal and TRPM8, as previously reported (Ciobanu et al., 2016).

Air–liquid interface (ALI) exposures to acetaldehyde or methylglyoxal also disrupted cytoskeletal organization in EpiAirway™ tissues, as indicated by Gene Ontology (GO) terms related to cadherin binding, focal adhesion, and cell–substrate junctions, and by predictions from Ingenuity Pathway Analysis (IPA). IPA further identified impaired cell spreading and lamellipodia formation, findings supported by BEAS-2B submerged culture experiments, which showed decreased stabilized f-actin and increased cell rounding which was concentration-dependent. Notably, cytoskeletal destabilization and blebbing, particularly in methylglyoxal-treated cells, were attenuated by antagonists of TRPA1 or TRPM8, implicating partial involvement of these TRP channels in the cytoskeletal responses to aldehyde exposure.

*In vivo*, acetaldehyde and methylglyoxal are produced during normal metabolic processes (Allaman et al., 2015; Takeuchi et al., 2024), and most organisms have enzymatic machinery to quickly convert both chemicals into less toxic products (Xue et al., 2011; Maessen et al., 2015). Acetaldehyde, a byproduct of alcohol metabolism, is broken down into acetate, a less toxic chemical (Sapkota and Wyatt, 2015). Methylglyoxal is produced during glycolysis and is eliminated via the glyoxalase system (Schalkwijk and Stehouwer, 2020; He et al., 2020), which catalyzes its conversion to a less toxic product (D-lactate) plus glutathione (Rabbani and Thornalley, 2011; Rabbani and Thornalley, 2014; Chakraborty et al., 2014).

However, excess exposure to acetaldehyde or methylglyoxal can cause clinically significant effects. Acetaldehyde has been implicated in the progression of tobacco-related diseases due to its high concentrations in cigarette smoke (0.6–2 mg/cigarette) (Sapkota and Wyatt, 2015; Uchiyama et al., 2013). Acetaldehyde generates protein adducts in lungs of smokers (Wyatt et al., 2012; Sapkota and Wyatt, 2015) and may contribute to chronic obstructive pulmonary disease (COPD) by causing mitochondrial dysfunction and inflammation in airway epithelial cells (Kawano et al., 2004; Wyatt et al., 2012; Li et al., 2019). Acetaldehyde also causes bronchoconstriction (Kawano et al., 2004), and has been linked to cardiovascular diseases (Kupari et al., 1983; Zhang J. et al., 2023), cancer (Acetaldehyde, 2009; Hanssen et al., 2014; Seitz et al., 2009) and enhancing nicotine addiction (Talhout et al., 2007). In excessive drinkers, acetaldehyde contributes to alcoholic liver disease through inflammation, adduct formation, and DNA damage (Appelman et al., 1982; Setshedi et al., 2010; Yan et al., 2023). Excess exposure to methylglyoxal is linked to ageing, diabetes, obesity, atherosclerosis, hypertension, cancer, and neuropathies (Matafome et al., 2013; Bellahcène et al., 2018; Bellier et al., 2019; Schalkwijk and Stehouwer, 2020; Maessen et al., 2015; Rabbani and Thornalley, 2011; Rabbani and Thornalley, 2014; Hanssen et al., 2014). Methylglyoxal is elevated in the blood of diabetics and is involved in diabetic pathologies, as well as in AGE-related diseases (Bellier et al., 2019; Schalkwijk and Stehouwer, 2020; Mukohda et al., 2012; Shamsaldeen et al., 2016).

At the cellular level, pathogenesis induced by these aldehydes is largely mediated through the formation of adducts. Both acetaldehyde and methylglyoxal generate DNA adducts (Setshedi et al., 2010; Sapkota and Wyatt, 2015; Schalkwijk and Stehouwer, 2020; Chen et al., 2007; Nissen et al., 2022), which impair DNA repair mechanisms (Barry, 1988; Setshedi et al., 2010; Wyatt et al., 2012; Chen et al., 2007; Lai et al., 2022; Wang et al., 2000; Brooks and Theruvathu, 2005), promote mutagenesis, and potentially contribute to carcinogenesis (Bellier et al., 2019; Brooks and Theruvathu, 2005; Mizumoto et al., 2017). In addition, both aldehydes form protein adducts that disrupt critical cellular functions, including signal transduction and apoptosis regulation, ultimately leading to aberrant cell signaling or cell death (Setshedi et al., 2010; Conduah et al., 1998; Jokelainen et al., 1998; Jokelainen et al., 2000). These compounds also elevate intracellular reactive oxygen species (ROS), exacerbating oxidative stress and further promoting adduct formation (Yan et al., 2023; Takeuchi et al., 2024; Kim et al., 2004; Ramasamy et al., 2006; Novitskiy et al., 2006; Desai et al., 2010; Tamura et al., 2014).

Methylglyoxal also forms AGEs through reactions with proteins that are associated with cellular dysfunction, age-related pathologies, and neurodegenerative diseases (Ramasamy et al., 2006; Raghavan, 2024; Dhar and Desai, 2012). Consistent with these mechanisms, proteomic analysis of EpiAirway™ tissues exposed to either aldehyde revealed changes in pathways associated with DNA repair, G2/M DNA damage checkpoint regulation, fatty acid metabolism (Figures 1C, 2A,B), and protein translation (Figure 3), as indicated by Gene Ontology (GO) and Ingenuity Pathway Analysis (IPA). The well-documented capacity of both acetaldehyde and methylglyoxal to form adducts with DNA, proteins, and lipids likely underlies the observed effects on these essential cellular processes.

Limitations: Our purpose was to characterize the toxicity of acetaldehyde and methylglyoxal when tested in isolation. However, other EC constituents, puffing topography which varies with users (Kośmider et al., 2018; Behar et al., 2015), and EC design which is continually evolving (Hsu et al., 2018; Trtchounian and Talbot, 2011; Williams et al., 2019) could affect the levels and toxicity of these aldehydes. As an example, pretreatment of osteoblastic cells with limonene, a flavor chemical sometimes added to EC fluids (Omaiye et al., 2019), prevented methylglyoxal induction of cell death and reduced endoplasmic reticulum stress, autophagic activity, and ROS (Suh et al., 2017). Conversely, other related toxic chemicals, such as glyoxal and formaldehyde, are produced during vaping (Jensen et al., 2017; Uchiyama et al., 2020; Saliba et al., 2018), and these may interact additively or synergistically with acetaldehyde and methylglyoxal to increase their toxicity.

## 5 Conclusion

This study introduces a novel approach to comparative toxicology by evaluating the relative toxicities of acetaldehyde and methylglyoxal and identifying shared molecular targets. Proteomic analysis of 3D EpiAirway™ tissues exposed at the ALI provided a sensitive and informative platform for comparing these two aldehydes. More than 65% of the DEPs overlapped between chemicals, with strong concordance in response profiles, including direction and magnitude of fold changes at exposure levels relevant to EC users. Both acetaldehyde and methylglyoxal adversely affected biological, cellular, and molecular processes at concentrations reported in EC aerosols.

Functional assays with BEAS-2B cells supported and extended the proteomics findings, demonstrating that mitochondria and the cytoskeleton are primary targets of both aldehydes, accompanied by increased reactive oxygen species (ROS) generation. Notably, methylglyoxal elicited these effects at concentrations approximately 200-fold lower than acetaldehyde in both ALI and submerged exposure models. Some toxicological effects were mediated through activation of TRPA1 and TRPM8 channels, suggesting mechanistic involvement of transient receptor potential (TRP) signaling pathways.

The potency of both aldehydes at low concentrations raises concern about their potential to contribute to EC-induced tissue injury. These findings underscore the need for further toxicological investigation into aldehydes such as methylglyoxal, which, despite being present at lower concentrations than more extensively studied compounds like acetaldehyde and diacetyl, may exert substantial biological effects.

## Data Availability

Raw data used for the study is provided in the Supplementary Material for download. All proteomics data presented in the study were derived from the raw proteomics excel file (Supplementary Table S3). This file contains uniprot ID, gene name, log2 fold change values, p-values, and adjusted p-values. Significant proteins (adj p < 0.05) were used for GO enrichment analysis and IPA analysis.

## Supplementary material

SUPPLEMENTARY FIGURE 1 Treemap plots for GO Molecular functions. Treemap plots for acetaldehyde **(A)** and methylglyoxal **(B)**. GO Terms were clustered together based on similarity using the rrvgo package. Each cluster has one parent GO Term that describes the cluster and is bold and darker in color than other terms in the cluster. Within individual treemaps, box size corresponds to significance (–log10(p-value)) with more significant terms having larger sized boxes. Treemaps are not scaled across **(A,B)**. Overlapping clusters between the two groups are colored alike and asterisks indicate parent GO Terms found in both groups. **(C)** Circle plot connecting proteins to Unique GO Terms from Molecular Function ontology found only in methylglyoxal treated EpiAirway™. The color scale indicates the magnitude of the fold change for different proteins.

SUPPLEMENTARY FIGURE 2 Circle plot connecting proteins to Unique GO Terms from Biological Process ontology found only in methylglyoxal treated EpiAirway™. Protein names in black were exclusive to methylglyoxal, while protein names in grey were in both methylglyoxal and acetaldehyde treated EpiAirway™. The scale bar shows the magnitude of the fold change for different proteins.

SUPPLEMENTARY TABLE 1 Acetaldehyde concentrations reported in the literature. All values are converted into ug/mL to allow for comparison across studies.

SUPPLEMENTARY TABLE 2 Methylglyoxal concentrations reported in the literature. All values are converted into ug/mL to allow for comparison across studies.

SUPPLEMENTARY TABLE 3 Proteomics File. Excel file containing abundance ratios, log2 fold changes, adjusted p-values, and p-values of all the proteins from the TMT proteomics analysis.

## Abbreviations

ALI, Air-liquid-interfac; EC, Electronic cigarette; TRP, Transient receptor potential; BEGM, Bronchial epithelial cell growth medium; TMT, Tandem Mass Tag; IPA, Ingenuity Pathway Analysis; GO, Gene Ontology; MTT, 3-(4,5-dimethylthiazol-2-yl)-2,5-diphenyltetrazolium bromide; DEP, Differentially expressed protein; 3 D human bronchial epithelial tissue, EpiAirway™; AGE, Advance glycation end product; ROS, Reactive oxygen species.
