# Supplementary material for: Acetaldehyde and methylglyoxal: comparative analysis of toxic electronic cigarette degradation products in 3D and 2D exposure systems using human bronchial epithelial models
Source: Front Toxicol. 2025 Sep 30;7:1624794. doi: 10.3389/ftox.2025.1624794 (PMC12518343; doi:10.3389/ftox.2025.1624794)

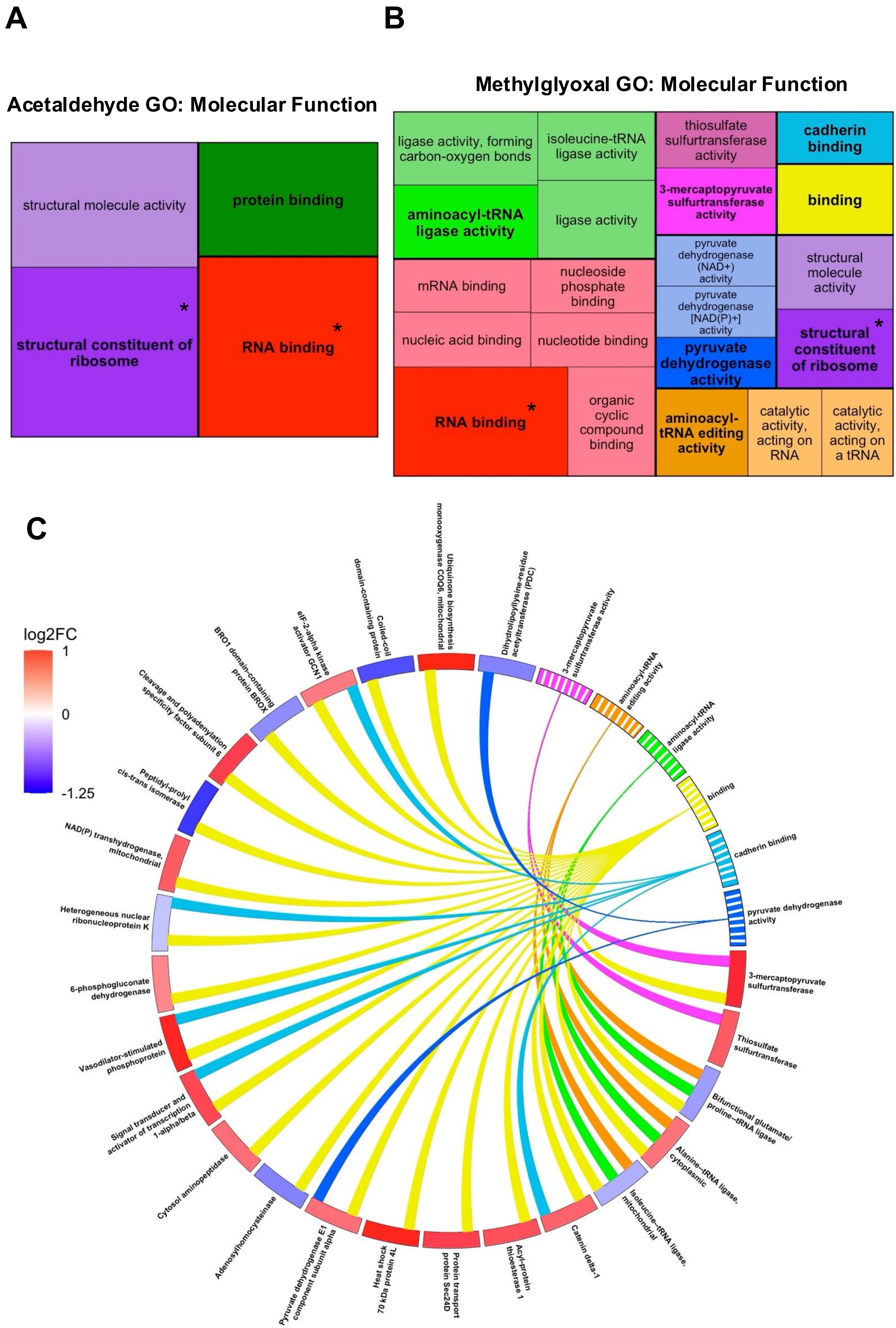
A
Acetaldehyde GO: Molecular Function
structural molecule activity
protein binding
structural constituent of ribosome
*
RNA binding
*
B
Methylglyoxal GO: Molecular Function
ligase activity, forming carbon-oxygen bonds
isoleucine-tRNA ligase activity
thiosulfate sulfurtransferase activity
cadherin binding
aminoacyl-tRNA ligase activity
ligase activity
3-mercaptopyruvate sulfurtransferase activity
binding
pyruvate dehydrogenase (NAD+) activity
structural molecule activity
mRNA binding
nucleoside phosphate binding
pyruvate dehydrogenase [NAD(P)+] activity
nucleic acid binding
nucleotide binding
pyruvate dehydrogenase activity
structural constituent of ribosome
*
RNA binding
*
organic cyclic compound binding
aminoacyl-tRNA editing activity
catalytic activity, acting on RNA
catalytic activity, acting on a tRNA
C
log2FC
1
0
-1.25
Ubiquinone biosynthesis monooxygenase COQ6, mitochondrial
Coiled-coil domain-containing protein
eIF-2-alpha kinase activator GCN1
BRO1 domain-containing protein BROX
Cleavage and polyadenylation specificity factor subunit 6
Peptidyl-prolyl cis-trans isomerase
NAD(P) transhydrogenase, mitochondrial
Heterogeneous nuclear ribonucleoprotein K
6-phosphogluconate dehydrogenase
Vasodilator-stimulated phosphoprotein
Signal transducer and activator of transcription 1-alpha/beta
Cytosol aminopeptidase
Adenosylhomocysteinase
Pyruvate dehydrogenase E1 component subunit alpha
Heat shock 70 kDa protein 4L
Protein transport protein Sec24D
Acyl-protein thioesterase 1
Catenin delta-1
Isoleucine--tRNA ligase, mitochondrial
Alanine--tRNA ligase, cytoplasmic
Bifunctional glutamate/proline--tRNA ligase
Thiosulfate sulfurtransferase
3-mercaptopyruvate sulfurtransferase
pyruvate dehydrogenase activity
cadherin binding
binding
aminoacyl-tRNA ligase activity
aminoacyl-tRNA editing activity
3-mercaptopyruvate sulfurtransferase activity
Dihydrolipoyllysine-residue acetyltransferase (PDC)

GO Biological Process: Methylglyoxal

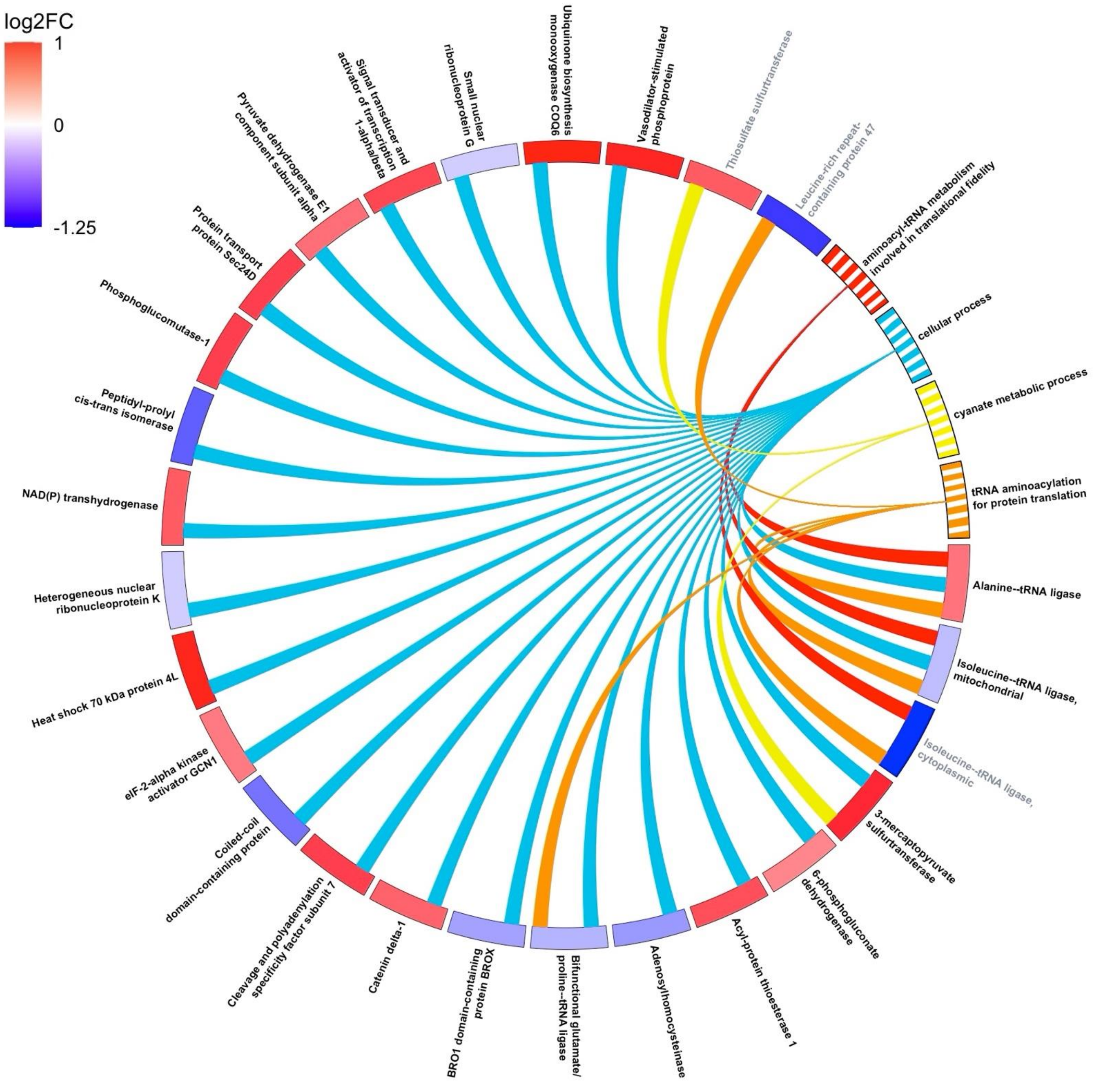

Supplement: Supplementary file 4 [file Image1.pdf]
